# Assessing the Cost of Healthy and Unhealthy Diets: A Systematic Review of Methods

**DOI:** 10.1007/s13668-022-00428-x

**Published:** 2022-09-09

**Authors:** Cherie Russell, Jillian Whelan, Penelope Love

**Affiliations:** 1 School of Exercise and Nutrition Sciences, Deakin University, Geelong, Australia; 2 School of Medicine, Institute for Health Transformation, Deakin University, Geelong, Australia; 3 Institute for Physical Activity and Nutrition, Deakin University, Geelong, Australia

**Keywords:** Food pricing, Food cost, Measurement instruments, Food environments

## Abstract

**Purpose of Review:**

Poor diets are a leading risk factor for chronic disease globally. Research suggests healthy foods are often harder to access, more expensive, and of a lower quality in rural/remote or low-income/high minority areas. Food pricing studies are frequently undertaken to explore food affordability. We aimed to capture and summarise food environment costing methodologies used in both urban and rural settings.

**Recent Findings:**

Our systematic review of high-income countries between 2006 and 2021 found 100 relevant food pricing studies. Most were conducted in the USA (*n* = 47) and Australia (*n* = 24), predominantly in urban areas (*n* = 74) and cross-sectional in design (*n* = 76). All described a data collection methodology, with just over half (*n* = 57) using a named instrument. The main purpose for studies was to monitor food pricing, predominantly using the ‘food basket’, followed by the Nutrition Environment Measures Survey for Stores (NEMS-S). Comparatively, the Healthy Diets Australian Standardised Affordability and Price (ASAP) instrument supplied data on relative affordability to household incomes.

**Summary:**

Future research would benefit from a universal instrument reflecting geographic and socio-cultural context and collecting longitudinal data to inform and evaluate initiatives targeting food affordability, availability, and accessibility.

**Supplementary Information:**

The online version contains supplementary material available at 10.1007/s13668-022-00428-x.

## Introduction

Poor diets, described as those low in fruits, vegetables, and whole grains, and high in red and processed meats and ultra-processed foods, are a leading risk factor for chronic disease globally [1]. In most high-income countries (HIC), poor diets disproportionally affect lower socioeconomic populations, Indigenous Peoples, and those living in rural and/or remote areas [2–5]. Rather than solely a consequence of individual behaviours, poor diets are critically informed by broad contextual factors, including social, commercial, environmental, and cultural influences [6, 7]. Crucially, the consumption of a healthy diet is constrained by the range, affordability, and acceptability of foods available for sale [8]. Research suggests that healthy foods are often harder to access, more expensive, and often of a lower quality in rural, remote, or low-income/high minority areas, than in metropolitan or high-income areas [9–12]. Such food environments contribute to higher rates of diet-related non-communicable diseases and food insecurity [13, 14]. In order to improve population diets, all aspects of the food environment must be addressed to ensure healthy foods are affordable, available, and of adequate nutritional quality [15].

Price is a primary factor impacting food choice, diet quality, and food security, therefore having affordable, acceptable, healthy food should be a political and social priority [8, 15, 16]. Some research suggests that healthy diets are associated with greater total spending [17–19], while other studies report that adherence to a healthy diet is less expensive than current or ‘unhealthy’ diets [9, 20, 21]. Regardless, the cost of a healthy diet is a proportionately large household expense (> 30% of household income) and may therefore be considered ‘unaffordable’ [22]. Additionally, public perception that healthy diets are expensive is high, which itself may be a barrier to the purchase of healthy foods [23]. Therefore, improving the affordability of healthy food could improve population diets, regardless of context [24].

To address the issue of food affordability and inform appropriate attenuating policy and intervention strategies, food pricing studies are frequently undertaken. Food pricing, however, is not a universal construct and is highly influenced by country and context. Numerous methods have been developed to measure food pricing, with data therefore not always comparable or replicable, and of limited value to inform appropriate policy [25]. Most studies that collect food pricing data conclude that food prices are rising, making healthy eating unaffordable for many populations. However, few studies to date have used this data to suggest strategies to improve affordability. Our systematic review aims to capture and summarise food environment costing methodologies used in HIC, in both urban and rural settings, between 2006 and 2021. In addressing this aim, we answer the following questions: (i) What is the stated purpose of collecting data on food prices, including whether the data is used to inform or advocate for interventions? (ii) Which instruments are being used to measure food pricing? (iii) What are the strengths and limitations of each instrument as reported by study authors?

## Methods

To address the research aim, we undertook a systematic review of the literature, following the Preferred Reported Items for Systematic Reviews and Meta-Analyses (PRISMA) guidelines [26]. We followed four steps: (i) systematic search for relevant literature; (ii) selection of studies, (iii) data extraction, and (iv) analysis and synthesis of results.

### Systematic Search Strategy

After consultation with a research liaison librarian, databases used included *EBSCOHOST* (Academic Search Complete, CINAHL Complete, GlobalHealth, Medline Complete, and PsychINFO) and *Informit.* We chose these databases for their comprehensiveness and conventional use in the public health nutrition discipline. We identified search terms using a scoping review and key words used in previous food pricing reviews [15, 23, 27, 28]. We searched both article abstracts and titles using the following search string: ‘food affordability’ OR ‘food cost’ OR ‘food price*’ OR ‘food promotion*’. We completed an initial search for studies published 2016–2021 in October 2021, followed by a search for studies published 2006–2015 in December 2021.

### Selection of Studies

Studies were included if they were English, peer-reviewed journal articles presenting original research, monitored food prices in a high-income country/s, and were published between 2006 and 2021. The article by Glanz (2006) [15] is considered a seminal paper in food pricing research and was therefore chosen as the starting date for our search. Studies prior to this date were considered unlikely to be relevant to the research question and were thus excluded. Review articles, opinion pieces, posters, perspectives, study protocols, viewpoints, editorials, and commentaries were excluded, as well as those assessing middle- or low-income countries.

Study screening involved an initial review of all titles and removal of duplicates by A1 using online *Covidence* software [29], followed by abstract screening (A1), and then full text screening of remaining studies (A1). A second reviewer independently screened all articles by abstract and full text to minimise bias (A2 and A3). Disagreements were resolved through discussion between researchers; where no agreement was reached, a third party acted as an arbiter (A2 and A3). Limited hand searching was conducted given the volume of papers identified. Online Resource 1 presents a PRISMA flow chart of the study selection process.

### Data Extraction

Included studies were uploaded to an *Endnote* (V. X9) [30] library. We systematically extracted details of each study to *Microsoft Excel* (V. 2112), including the author/s, year published, article title, aim, pricing instrument used (if specified), country and geographical context (e.g. urban or rural), type of data collected, number and type of locations assessed, number and type of food items captured, population (if the study used sales receipts to estimate food prices), time period of study, strengths, limitations, and conclusions.

### Data Analysis and Synthesis

The coded data were used to identify major themes that were then synthesised in the results. We used an inductive thematic approach for our analysis, with the results discussed between the research team to limit researcher subjectivity [31]. We used *Microsoft Excel* to calculate descriptive statistics and graphical outputs.

## Results

### Overview of Studies

Database searching identified 2737 studies, with 1882 studies remaining after removal of duplicates. After abstract screening, a total of 287 were identified for full-text screening, with 187 excluded, and a total of 100 studies included in this systematic review (Online Resource 1).

**Fig. 1:**
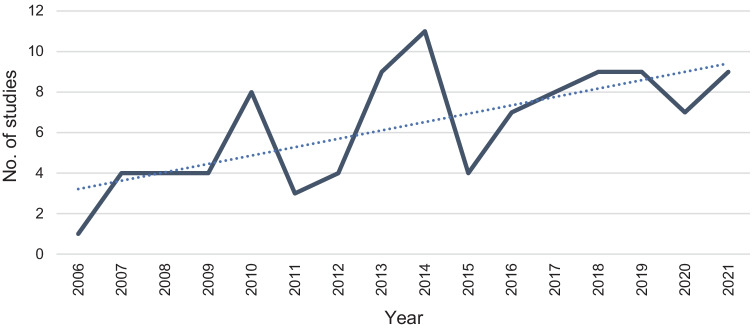
Frequency of studies published assessing food prices between 2006 and 2021

We observed an increasing number of studies each year, with peaks in 2013, 2014, and 2018 (Fig. 1).

Most studies measured food prices in the USA (*n* = 47), followed by Australia (*n* = 25). Urban food environments were assessed more frequently (*n* = 74) than rural (*n* = 33). Most studies were cross-sectional (*n* = 77). Most studies included instore price audits (*n* = 59), followed by online price audits (supermarket websites, *n* = 13), or electronic point of sale data (consumer receipts, register sales, or electronic scanning of food prices in the home, *n* = 12), and a combination of these (*n* = 17). Most studies collected food price data from more than 20 food retail outlets (*n* = 34) (Table 1).

**Table 1:** Overview of studies (*n* = 99); *n* > total number of included studies as some studies looked at multiple characteristics

Characteristic		*N*
Country	USA [11, 32–75]	47
	Australia [9, 10, 19, 20, 76–94]	25
	UK [95–102]	8
	Canada [103–109]	7
	New Zealand [110–113]	4
	Germany [71, 114, 115]	3
	Portugal [116–118]	3
	Netherlands [119, 120]	2
	Sweden [121, 122]	2
	Japan [123]	1
	Croatia [124]	1
	Demark [125]	1
	France [126]	1
Context	Urban [20, 33, 36, 38, 39, 41–48, 53–55, 57–60, 62–72, 75–77, 79, 80, 86, 89, 92, 95, 98, 99, 102, 104, 105, 108, 112, 114–117, 119, 121, 124–126]	55
	Mixed urban/rural [19, 32, 37, 40, 50, 52, 61, 73, 81, 83–85, 88, 94, 96, 97, 110, 111, 113, 123, 127]	20
	Rural [9–11, 56, 78, 82, 87, 90, 91, 103, 106, 109]	13
	Not specified [34, 35, 49, 51, 74, 93, 100, 101, 107, 120, 122]	11
Study design	Cross-sectional [9–11, 20, 32, 35, 36, 39, 40, 42–47, 49, 51–57, 59, 60, 62, 64, 67–73, 75–79, 81, 82, 84–89, 91, 92, 94–96, 99–110, 112–117, 119–122, 124, 125]	76
	Longitudinal [19, 33, 34, 37, 38, 41, 48, 50, 58, 61, 63, 65, 66, 74, 80, 83, 90, 93, 97, 98, 111, 123, 127]	24
Data sources	Instore price audit [9–11, 19, 20, 32, 33, 36, 38, 39, 42–44, 46–52, 54–57, 59, 60, 66–73, 75, 77, 79, 80, 83, 85–93, 98, 102–110, 112, 114, 115, 119–121, 124]	65
	Electronic point of sale data [34, 37, 40, 41, 45, 61, 74, 76, 78, 82, 94, 96, 97, 111, 126, 127]	16
	Online price audit [35, 53, 58, 65, 95, 99–101, 116, 123]	10
	Instore and online price audits [62–64, 81, 84, 113, 117, 122, 125]	9
No. of food retail outlets	One [53, 55, 58, 95, 99, 116]	6
	Between 2 and 10 [11, 20, 62–65, 72, 74, 75, 79, 84, 103, 105, 113, 117, 119–125]	22
	Between 11 and 20 [10, 38, 46, 51, 66, 70, 71, 82, 89, 91, 98, 104, 109, 112]	14
	More than 20 [9, 19, 33, 36, 37, 39, 40, 42–44, 47–49, 52, 54, 56, 57, 59, 60, 68, 73, 80, 81, 83, 87, 88, 92, 94, 106–108, 110, 111, 115, 127]	35
	Not specified [32, 34, 35, 41, 45, 50, 61, 67, 69, 76–78, 85, 86, 90, 93, 96, 97, 100–102, 114, 126]	23
Named instrument used	Yes [9–11, 19, 20, 33, 36–40, 43, 44, 46–52, 54–57, 59, 60, 62–64, 66–68, 70, 71, 73, 76, 80, 81, 83, 85–92, 94, 101, 103–108, 110, 111]	57
	No [32, 34, 35, 41, 42, 53, 58, 61, 65, 69, 72, 74, 75, 77–79, 82, 84, 93, 95–100, 102, 109, 112–127]	43
Method used	Healthy food basket varieties [9, 19, 20, 37, 38, 40, 46, 49–52, 62–64, 66, 67, 70, 80, 81, 83, 85–92, 94, 103–105, 110]	31
	NEMS-S and variants [10, 11, 33, 36, 44, 47, 48, 54, 56, 57, 68, 71, 106–108]	15
	Other [32, 34, 35, 39, 41–43, 53, 55, 58–61, 65, 69, 72–79, 82, 84, 93, 95–102, 109, 111–127]	52

*NEMS-S*, Nutrition Environment Measures Survey-Stores

Details of all included studies, grouped according to data source used (instore price audits, online price audits, electronic point of sale, and combinations of these), are shown in Tables 2, 3, and 4. Details include instrument used (if applicable), purpose of data collection, country, context, study type (e.g. cross-sectional, longitudinal), healthiness comparisons (between healthy and unhealthy products or diets), study author, and year. The use of a named instrument was captured to identify commonalities in usage of instruments, and not as an indication of study quality. When assessing differentials in ‘healthiness’, studies either presented a comparison of a ‘healthy diet’ with an ‘unhealthy or currently consumed diet’ or a comparison of the cost of ‘healthy’ and ‘unhealthy’ foods or product categories.

**Table 2:** A summary of studies measuring food prices using instore price audits; *n* > total number of included studies as some studies looked at multiple characteristics

Named instrument used (if applicable) [country of origin]	Purpose of data collection	Country (*n*)	Context (*n*)	Study type (*n*)	Healthiness comparison	Study references
**Instore food price audits (*n* = 59 studies)**						
Victorian Healthy Food Basket (VHFB) [Australia]	- Monitor food prices in an area [80] - Examine factors related to changing food prices [80, 87] - Assess association of distance and socioeconomic status with food prices [19, 91]	Australia – 4	Urban – 3 Rural – 2	Cross-sectional – 2 Longitudinal – 2	Product category – 2 No -2	Palermo et al.’16 [19] Cuttler et al.’19 [80] Palermo et al.’08 [87] Ward et al.’12 [91]
Healthy Food Access Basket Survey (QLDHFAB) [Australia]	- Monitor food cost over time [83] - Monitor food cost by location [88]	Australia – 2	Urban – 2 Rural – 1	Cross-sectional – 1 Longitudinal – 1	Diet – 1 Product category – 1	Harrison et al.’07 [83] Pollard et al.’14 [88]
The Illawarra Healthy Food Basket (IHFB) survey [Australia]	- Compare food price by location [89] - Assess relative food price for welfare recipients over time [90]	Australia – 2	Rural – 1 Urban – 1	Cross-sectional – 1 Longitudinal – 1	Product category – 2	Tsang et al.’07 [89] Walton et al.’21 [90]
Adelaide Healthy Food Basket [Australia]	- Compare food price by location [89] (Using adapted Illawarra healthy food basket) - Assess impact of rurality and SES on food price [92]	Australia – 2	Urban – 1 Rural – 1	Cross-sectional – 2	Product category – 1 No – 1	Tsang et al.’07 [89] Wong et al.’11 [92]
Food basket informed by the INFORMAS framework [Australia]	- Compares price of healthy and current diets for different incomes [20, 110]	Australia – 1 New Zealand – 1	Urban – 2 Rural – 1	Cross-sectional – 2	Diet – 2	Lee et al.’16 [20] Mackay et al.’18 [110]
Healthy Diets Australian Standardised Affordability and Price (ASAP) Survey [Australia]	- Compares price of healthy and current diets for different incomes [9, 85, 86]	Australia – 3	Urban – 2 Rural – 2	Cross-sectional – 3	Diet – 3	Love et al.’18 [9] Lee et al.’21 [85] Lee et al.’20 [86]
University of Washington’s (UW) Center for Public Health Nutrition (CPHN) market basket [USA]	- Assess impact of government welfare/minimum wage on the ability to buy healthy food [38, 66, 70]	USA – 3	Urban – 3	Cross-sectional – 1 Longitudinal – 2	Product category – 2 No – 1	Buszkiewicz et al.’19 [38] Otten et al.’17 [66] Spoden et al.’18 [70]
USDA Market Basket [USA]	- Measure association between food cost, nutritional quality and location [50, 51]	USA – 2	Urban – 2 Rural – 1	Cross-sectional – 1 Longitudinal – 1	Product category – 1 No – 1	Hardin-Fanning and Rayens’15 [50] Hardin-Fanning and Wiggins’17 [51]
USDA Authorized Food Retailers’ Characteristics and Access Study [USA]	- Measure food costs and compare between food groups	USA	Urban	Cross-sectional	Product category	Connell et al.’12 [43]
USDA Low-cost food plan [USA]	- Measure differences between food cost, energy and nutritional quality	USA	Urban	Cross-sectional	No	Karp et al.’14 [55]
Based on previous price surveys conducted by the Hartford Advisory Commission on Food Policy [USA]	- Compare food costs between locations	USA	Urban	Cross-sectional	No	Martin et al.’14 [59]
Based on a tool developed by the Yale Rudd Center [USA]	- Measure price differential between small and large stores	USA	Urban	Cross-sectional	No	Caspi et al.’17 [39]
Flint Store Food Assessment Instrument [USA]	- Compare cost of food categories and locations	USA	Urban	Cross-sectional	No	Mayfield et al.’20 [60]
USDA Food Store Survey Instrument [USA]	- Assess food prices in a location	USA	Urban Rural	Cross-sectional	No	Wright et al.’18 [73]
Revised Northern Food Basket [Canada]	- Use food price data to develop public policy	Canada	Rural	Cross-sectional	Product category	Kenny et al.’18 [103]
Ontario Nutritious Food Basket [Canada]	- Compares food cost in different locations	Canada	Urban	Cross-sectional	No	Latham and Moffat’07 [104]
Unspecified Food Basket [Canada]	- Assess association of food prices and BMI	Canada	Urban	Cross-sectional	No	Lear et al.’13 [105]
Nutrition Environment Measures Survey – Stores (NEMS-S) * [including NEMS-S-Rev, TxNEAS and NEMS-S-NL, Bridging the Gap Food Store Observation Form] [USA]	- Assess food prices as a function of income [33, 36] - Monitor food prices in an area [33, 54, 56, 57, 106, 108] - Compare perceptions with actual food prices [68, 107] - Identify intervention strategies/policies to improve public health [10, 11] - Compare food prices between countries [71] - Understand impact of pricing strategies on consumption behaviours [44] - Correlate food price with obesity [47] - Measure impact of opening a store in a food desert on food prices [48]	USA – 11 Canada – 3 Australia – 1 Germany—1	Rural – 4 Urban – 11	Cross-sectional – 13 Longitudinal – 2	Diet – 15	Whelan et al.’18 [10] Pereira et al.’14 [11] Andreyeva et al.’08 [33] Borja and Dieringer’19 [36] DiSantis et al.’14 [44] Ghosh-Dastidar et al.’14 [47] Ghosh-Dastidar et al.’17 [48] Jin and Lu’21 [54] Ko et al.’18 [56] Lee Smith et al.’13 [57] Shen et al.’19 [68] Stroebele-Benschop et al.’20 [71] Mah et al.’20 [106] Minaker et al.’13 [107] Minaker et al.’14 [108]
n/a	- Assess food cost in relation to geographic distance and socioeconomic status	Australia	Urban	Cross-sectional	Product category	Ball et al.’09 [77]
n/a	- Assess the price of healthy and unhealthy food over time	Australia	Not specified	Longitudinal	Diet and product category	Burns et al.’08 [93]
n/a	- Assess prices of branded and generic products	Australia	Urban	Cross-sectional	Product category	Chapman et al.’13 [79]
n/a	- Assess the cost of a healthy and unhealthy diet	New Zealand	Urban	Cross-sectional	Diet	Vandevijvere et al.’18 [112]
n/a	- Assess food prices in specific locations	USA	Urban	Cross-sectional	No	Cole et al.’10 [42]
n/a	- Assess impact of external factors on diet cost in homeless families	USA	Urban	Cross-sectional	Product category	Smith et al.’10 [69]
n/a	- Assess food cost and diet quality	USA	Urban	Cross-sectional	Diet	Townsend et al.’09 [72]
n/a	- Assess food prices in a location	USA	Urban	Cross-sectional	No	Zenk et al.’10 [75]
n/a	- Assess food prices in a location	Canada	Rural	Cross-sectional	Product category	Pakseresht et al.’14 [109]
n/a	- Assess association between food cost and diet	UK	Urban	Cross-sectional	Diet and product category	Vogel et al.’19 [102]
n/a	- Assess food cost in relation to geographic distance	UK	Urban	Cross-sectional	Diet	Mackenbach et al.’17 [98]
n/a	- Assess association between food prices and diet quality	Netherlands	Urban	Longitudinal	Diet	Mackenbach et al.’19 [119]
n/a	- Assess association between food cost and energy density	Netherlands	Not specified	Cross-sectional	Diet and product category	Waterlander et al.’10 [120]
n/a	- Assess food prices in a location	Germany	Urban	Cross-sectional	Product category	Alexy et al.’12 [114]
n/a	- Assess food prices as a function of socio-economic status	Germany	Urban	Cross-sectional	Product category	Stroebele et al.’11 [115]
n/a	- Compare the cost of healthy and unhealthy food in relation to health status	Croatia	Urban	Cross-sectional	Product category	Bolarić and Šatalić’13 [124]
Market Basket Survey conducted by the NT Government [Australia]	- Measure price differential between Indigenous and capital city supermarkets	Australia	Urban – 1 Rural – 1	Cross-sectional – 1	No	Ferguson et al.’16 [81]
Market basket developed by Fred Hutchinson Cancer Research Center [USA]	- Compare prices of healthy and unhealthy foods [62] -Measure price of healthy food over time [63] - Assess food costs in an area [64]	USA – 3	Urban – 3	Cross-sectional – 2 Longitudinal – 1	Product category – 2 No -1	Monsivais and Drewnowski’07 [62] Monsivais et al.’10 [63] Monsiviais et al.’13 [64]
n/a	- Assess food price as a function of government welfare	Australia	Urban and rural	Cross-sectional	Product category	Kettings et al.’09 [84]
n/a	- Develop solutions for food prices for healthy diets	New Zealand	Urban and rural	Cross-sectional	Diet and product category	Wilson et al.’13 [113]
n/a	- Assess food prices in relation to socio-demographic factors and adherence to the Mediterranean diet	Portugal	Urban	Cross-sectional	Product category	Alves et al.’21 [117]
n/a	- Assess food basket prices in relation to health outcomes	Denmark	Urban	Cross-sectional	Product category	Parlesak et al.‘16 [125]
n/a	- Assess diet costs as a function of socio-economic status and diet quality	Sweden	Not specified	Cross-sectional	Product category	Rydén et al.’11 [122]
n/a	- Assess the cost of a healthy and current diet	Sweden	Urban	Cross-sectional	Product category	Rydén et al.’08 [121]

**Table 3:** A summary of studies measuring food prices using online price audits; *n* > total number of included studies as some studies looked at multiple characteristics

Named instrument used (if applicable) [country of origin]	Purpose of data collection	Country (*n*)	Context (*n*)	Study type (*n*)	Healthiness comparison	Study references
**Online food price audits (*n* = 12 studies)**						
Diet and Nutrition Tool for Evaluation (DANTE) [UK]	- Compare diet diaries and household receipts to determine food costs [100, 101]	UK – 2	Urban – 2	Cross-sectional – 2	No—2	Timmins et al.’15 [100] Timmins et al.’13 [101]
Healthy Diets Australian Standardised Affordability and Price (ASAP) Survey [Australia]	- Determine reliability of online food and beverage price data - Compare the cost of healthy and unhealthy diet in relation to socio-economic area	Australia	Urban and rural	Cross-sectional	Diet	Zorbas et al. [94]
n/a	- Assess cost associated with dietary diversity	UK	Urban	Cross-sectional	No	Conklin et al.’16 [95]
n/a	- Assess cost of DASH adhering diets	UK	Urban	Cross-sectional	Diet	Monsivais et al.’15 [99]
n/a	- Assess the cost of a dietary pattern protective from cardiovascular disease	USA	Not specified	Cross-sectional	Product category	Bernstein et al.’10 [35]
n/a	- Demonstrate the weakness of comparing food cost with energy density	USA	Urban	Longitudinal	Product category	Lipsky’09 [58]
n/a	- Assess association of diet cost and diet quality	USA	Urban	Longitudinal	Diet	Nansel et al.’16 [65]
n/a	- Assess food prices over time	USA	Urban	Cross-sectional	Product category	Hillen’21 [53]
n/a	- Assess food prices in relation to socio-demographic factors and adherence to the Mediterranean diet	Portugal	Urban	Cross-sectional	No	Albuquerque et al.’17 [116]
n/a	- Assess dietary cost of children	Portugal	Not specified	Cross-sectional	No	Faria et al.’16 [118]
n/a	- Compare food cost with dietary intakes	Japan	Urban and rural	Longitudinal	Diet and product category	Keiko et al.’17 [123]

**Table 4:** A summary of studies measuring food prices using either electronic point of sale data or a combination of data sources; *n* > total number of included studies as some studies looked at multiple characteristics

Named instrument used (if applicable) [country of origin]	Purpose of data collection	Country (*n*)	Context (*n*)	Study type (*n*)	Healthiness comparison	Study references
**Electronic point of sale (*n* = 12 studies)**						
Food Label Trial [Australia]	- Compare food prices of healthy and unhealthy food items - Assess impact of a labelling scheme on food cost	Australia	Urban	Cross-sectional	Product category	Abreu et al.’19 [76]
New Zealand Food Price Index [New Zealand]	- Measure relative change in price of healthier and less healthy foods over time	New Zealand	Urban and rural	Longitudinal	Product category	Mackay et al.’19 [111]
n/a	- Assess food cost and nutrient status	Australia	Rural	Cross-sectional	Diet and product category	Brimblecombe et al.’13 [78]
n/a	- Assess price in specific communities	Australia	Rural	Cross-sectional	No	Ferguson et al.’17 [82]
n/a	- Compare price of healthy and unhealthy food with blood sugar levels	USA	Not specified	Longitudinal	Product category	Anekwe et al.‘14 [34]
n/a	- Assess relationship between food price and poverty	USA	Urban	Longitudinal	No	Colabianchi et al.’21 [41]
n/a	- Assess cost of food prices in relation to diabetes	USA	Urban and rural	Longitudinal	Product category	Meyerhoefer et al.’10 [61]
n/a	- Assess food prices over time	USA	Urban and rural	Longitudinal	Diet	Ng et al.’14 [127]
n/a	- Assess food prices over time	USA	Not specified	Longitudinal	No	Yang and Leung’20 [74]
n/a	- Assess cost of healthy diets	UK	Urban and rural	Cross-sectional	Diet	Jones et al.’18 [96]
n/a	- Assess food prices over time	UK	Urban and rural	Longitudinal	Product category	Lan et al.’21 [97]
n/a	- Assess food prices and nutritional quality	France	Urban	Longitudinal	Diet and product category	Marty et al.’15 [126]
**Instore and online food price audits (*n* = 10 studies)**						
Market Basket Survey conducted by the NT Government [Australia]	- Measure price differential between Indigenous and capital city supermarkets	Australia	Urban – 1 Rural – 1	Cross-sectional – 1	No	Ferguson et al.’16 [81]
Market basket developed by Fred Hutchinson Cancer Research Center [USA]	- Compare prices of healthy and unhealthy foods [62] - Measure price of healthy food over time [63] - Assess food costs in an area [64]	USA – 3	Urban – 3	Cross-sectional – 2 Longitudinal – 1	Product category – 2 No – 1	Monsivais and Drewnowski’07 [62] Monsivais et al.’10 [63] Monsiviais et al.’13 [64]
n/a	- Assess food price as a function of government welfare	Australia	Urban and rural	Cross-sectional	Product category	Kettings et al.’09 [84]
n/a	- Develop solutions for food prices for healthy diets	New Zealand	Urban and rural	Cross-sectional	Diet and product category	Wilson et al.’13 [113]
n/a	- Assess food prices in relation to socio-demographic factors and adherence to the Mediterranean diet	Portugal	Urban	Cross-sectional	Product category	Alves et al.’21 [117]
n/a	- Assess food basket prices in relation to health outcomes	Denmark	Urban	Cross-sectional	Product category	Parlesak et al.‘16 [125]
n/a	- Assess diet costs as a function of socio-economic status and diet quality	Sweden	Not specified	Cross-sectional	Product category	Rydén and Hagfors’11 [122]
n/a	- Assess the cost of a healthy and current diet	Sweden	Urban	Cross-sectional	Product category	Rydén et al.’08 [121]
**Instore price audit and electronic point of sale (*n* = 6 studies)**						
Thrifty Food Plan Market Basket [USA]	- Assess impact of government welfare/minimum wage on the ability to buy healthy food, and the subsequent impact on health [37, 40, 70] - Measure food price in an area [46, 52, 67] - Use food price data to develop public policy [49]	USA – 6	Urban – 5 Rural – 3	Cross-sectional – 5 Longitudinal – 1	No – 6	Bronchetti et al.’19 [37] Christensen and Bronchetti’20 [40] Franzen and Smith’10 [46] Greenberg et al.’20 [49] Hilbert et al.’14 [52] Richards and Smith’06 [67] Spoden et al.’18 [70]
**Instore and online food price audits and electronic point of sale (*n* = 1 study)**						
n/a	- Compare approaches for estimating diet costs	USA	Urban and rural	Cross-sectional	No	Aaron et al.’13 [32]

### Study Purpose for Collecting Data on Food Prices

The studies included in this review had a multitude of aims (Tables 2, 3, and 4). While most studies were conducted solely to monitor food prices in a specific location/s [33, 39, 42, 46, 47, 52, 54, 56, 57, 59, 64, 67, 71, 75, 80, 81, 88, 89, 104, 106, 108, 109, 114], others aimed to monitor food price changes over time [53, 63, 74, 83, 93, 97, 111, 127], assess food prices as a function of income, socioeconomic status, or welfare assistance [9, 19, 20, 33, 36–38, 40, 41, 66, 69, 70, 77, 84–86, 90–92, 94, 100, 110, 115–117, 122]; assess food price in relation to geographic distance [19, 77, 91, 92, 94, 98]; compare perceptions of food price with actual food prices [68, 101, 107]; and relate food price with a health outcome [34, 35, 37, 40, 47, 58, 70, 72, 78, 105, 116, 117, 124, 125], compare the price of healthy or unhealthy foods/diets [9, 20, 34, 43, 50, 51, 55, 60–65, 76, 85, 86, 93–96, 99, 102, 110–112, 120, 121, 123, 124, 126], assess diet costs for a specific population [82, 118], compare food prices between brands [79], compare approaches for estimating dietary costs [32], or understand how prices impact consumption [44]. Only seven studies specifically aimed to collect data to inform policy strategies and/or community interventions to improve population health [10, 11, 49, 80, 87, 103, 113]. However, 26 studies did discuss their study findings on food price in relation to potential further action to improve food environments [9, 19, 20, 33, 36, 37, 40, 43, 47, 49, 50, 54, 55, 59, 63, 64, 81, 85–88, 103–105, 110]. Specific suggested strategies included those targeting individuals, such as education campaigns to promote healthy and more affordable food choices [9, 36, 43, 45, 49, 50, 55], and those targeting environmental changes, such as taxes on ‘unhealthy’ foods [33, 49, 85, 104, 110], subsidies and exemptions for ‘healthy’ foods [9, 20, 45, 62, 63, 85, 104, 110], vouchers for farmer’s markets [43], establishing more food stores [33, 45, 48, 104], better public transportation for consumers to access food stores [59], generating savings at the manufacturer/wholesaler level that can be passed on to customers [81], establishing community-led food supply options [9], and increasing welfare support proportionate to food prices and geographic distances to food stores [37, 40, 50, 73, 85].

### Overview of Instruments Used to Measure Food Prices

Of the 100 included studies, 57 used a named instrument to measure food prices, as described below. The remaining 43 studies did not name a pre-existing data collection instrument; instead, the authors described the data collection methodology used, for example, in store, online, or via electronic sales data.

#### Food Basket Instruments

The majority (*n* = 30) of studies used a variation of a ‘food basket’ to estimate food prices. Food baskets capture the prices of a pre-defined list of foods, often in quantities representative of the total diet of reference families over a defined timeframe [9], and is a longstanding methodology used to investigate the availability and affordability of food. Food basket studies were mainly conducted in the USA (*n* = 14) and Australia (*n* = 12) [19, 20, 80, 81, 83, 87–92]. Food basket studies using named instruments were conducted in the USA—using the Thrifty Food Plan Market Basket (*n* = 5), the Fred Hutchinson Cancer Research Center Market Basket (*n* = 3), the University of Washington’s Center for Public Health Nutrition Market Basket (*n* = 3), and the USDA Market Basket (*n* = 2); in Australia—using the Victorian Healthy Food Basket (*n* = 4), the Food Basket informed by the INFORMAS framework (*n* = 2), the Adelaide Healthy Food Basket (*n* = 2), the Illawarra Healthy Food Basket (*n* = 2), the Queensland Healthy Food Access Basket Survey (*n* = 1), and the Northern Territory Market Basket (*n* = 1); and in Canada—using the Ontario Nutritious food basket (*n* = 1), the Revised Northern Food Basket (*n* = 1), and an unspecified market basket (*n* = 1). Food basket studies were conducted in both rural (*n* = 13) [19, 37, 49, 50, 52, 81, 83, 87, 88, 90, 91, 103, 110] and urban contexts (*n* = 25) [19, 20, 37, 38, 40, 46, 49–52, 62–64, 66, 67, 70, 80, 81, 83, 88, 89, 92, 104, 105, 111].

All but two [37, 40] food basket studies collected prices from physical instore locations [19, 20, 38, 43, 46, 49–52, 55, 62–64, 66, 67, 70, 73, 80, 81, 83, 87–92, 103–105, 110], with four of these studies supplementing the data with online supermarket prices [62–64, 81]. Additionally, three instruments compared the cost of a ‘healthy diet’ to either an ‘unhealthy or currently consumed diet’ [20, 88, 110], 13 instruments compared the cost of ‘healthy’ and ‘unhealthy’ individual foods or product categories [19, 38, 51, 62, 63, 66, 83, 87, 89, 90, 103], and 14 instruments did not present a comparison [37, 40, 46, 49, 50, 52, 64, 67, 70, 80, 81, 91, 92, 104, 105]. ‘Current’ diets were defined using national survey data [20, 110]. Level of healthiness was defined using various benchmarks, namely the NOVA food processing classification system [38], nutrient composition and energy density [38, 51, 62, 63, 66, 80, 83, 90], national Dietary Guidelines [19, 43, 70, 87–90], and the Dietary Approaches to Stop Hypertension (DASH) dietary pattern [43]. Food affordability was benchmarked using household income [20, 49, 50, 90–92, 103, 105, 110], government subsidies [37, 40, 87, 89, 91], and minimum wage [38, 66, 70]; however, most studies (*n* = 13) did not determine relative affordability in their analysis [43, 51, 52, 55, 62–64, 67, 73, 80, 81, 83, 88].

#### Healthy Diets Australian Standardised Affordability and Price (ASAP) Instrument

Following critiques of existing food baskets, the previously described INFORMAS instrument was refined to assess and compare the price and affordability of healthy and current diets in Australia, leading to the development of the Healthy Diets Australian Standardised Affordability and Price (ASAP). This instrument assesses the cost of a ‘recommended’ Australian diet (defined by the Australian Dietary Guidelines and Australian Guide to Healthy Eating) and the cost of the ‘current’ Australian diet (as reported in the 2011–12 Australian Health Survey) using the reference household of two parents and two children (boy aged 14 years; girl aged 8 years) [128]. Thus, all studies using this instrument present a comparison of the cost of a ‘healthy’ and ‘unhealthy’ diet in their analysis. Intrinsic to the instrument, the relative affordability of a healthy diet is measured against household incomes. The ASAP instrument was used by four studies to collect food price data in physical instore locations [9, 85, 86] or from online supermarkets [94]. Studies were conducted in both rural (*n* = 2) [9, 85, 94] and urban (*n* = 2) [85, 86, 94] contexts.

#### Nutrition Environment Measures Survey for Stores (NEMS-S) Instrument

The Nutrition Environment Measures Survey for Stores (NEMS-S) and its variants were also frequently used throughout food pricing studies (*n* = 15). These included NEMS-S-Rev (Nutrition Environment Measures Survey for Stores Revised), TxNEAS (Texas Nutrition Environment Assessment), NEMS-S-NL (Nutrition Environment Measures Survey for Stores Newfoundland and Labrador), and The Bridging the Gap Food Store Observation Form. This instrument was used mostly in the USA (*n* = 11) [11, 33, 36, 44, 47, 48, 54, 57, 68, 71, 107]. Studies were conducted in both rural (*n* = 4) [10, 11, 56, 106] and urban (*n* = 11) [33, 36, 44, 47, 48, 54, 57, 68, 71, 107, 108] contexts. Compared to the food basket methodology, the NEMS-S instrument compares products in the same category that are considered ‘healthy’ or ‘unhealthy’ based on American Dietetic Association (ADA) recommended dietary guidelines, focusing on availability, price, and quality. All studies using the NEMS-S instrument collected food price data in physical instore locations. While the instrument itself does not include a calculation of relative affordability, approximately half the NEMS-S studies included this step in their methods [33, 36, 44, 47, 48, 54, 57], while all others did not [10, 11, 56, 68, 71, 106–108].

#### Other Instruments

Several other named instruments were identified, used in single studies. These included the Diet and Nutrition Tool for Evaluation (DANTE) [101], the Flint Store Food Assessment Instrument [60], the Food Label Trial registry tool [76], the New Zealand Food Price Index [111], the USDA Food Store Survey Instrument [73], USDA Low-cost food plan [55] and audit forms developed by the Yale Rudd Center [39], the Hartford Advisory Commission on Food Policy [59], and the USDA Authorized Food Retailers’ Characteristics and Access Study [43]. Only three instruments compared healthy and unhealthy products [43, 76, 111] and none analysed the relative affordability of food.

### Instrument Strengths and Limitations

The strengths and limitations of instruments commonly used across studies, as identified by study authors, are presented in Online Resource 2. Commonly cited limitations, regardless of instrument used, included that actual purchasing behaviours were not captured (unless electronic point of sales data was utilised); culturally important and region-specific products were often not captured; tools were cross-sectional in nature, thus seasonality or changes overtime were not considered; and out-shopping, described as food purchases undertaken outside the local residential geography, including internet orders or foods purchased during travel to other communities, could not be accounted for. While some food basket studies and those using the ASAP instrument did contextualise the relative affordability of healthy foods and/or diets, this was not a part of the methodology for NEMS-S. Other limitations specific to NEMS-S included the length of the survey, and a low convergence between NEMS-S results and consumer perceptions of affordability. Specific limitations for food basket studies included results being constrained by the reference family used and the assumption that food is shared equally among household members. Additionally, most instruments did not capture geographical information regarding access to food retail outlets or availability of foods within food retail outlets.

Authors less commonly described instrument strengths. For NEMS-S, cited strengths included the ability to compare food prices between healthy and unhealthy options, that it has strong inter-rater and test-re-test reliability, and that it has been validated in multiple countries. ASAP studies, and some food basket studies, included a comparison between healthy and current (‘unhealthy’) diets (based on actual consumption) and included alcohol in the survey.

## Discussion

Our systematic review details the key purposes, and methodologies used, for measuring food prices in HIC between 2006 and 2021. While most studies were conducted solely to monitor food prices in specific locations, some sought to report price changes over time, and others collected data to assess comparability of food costs to healthier alternatives, average earnings, welfare payments, rurality, and socioeconomic position. Most studies measured food prices in urban areas, using instore food price audits, with an emerging use of online data collection evident. The most frequently used instruments were ‘food baskets’, used predominantly to monitor food prices; the NEMS-S instrument, used to provide data on relative cost and availability; and the ASAP instrument, use to provide data on relative affordability.

Our review differs from previous reviews of food price and affordability instruments [23, 28] by taking a broadened focus on food pricing measures used in HIC globally and including new technology that is affording opportunities for electronic food pricing data collection. While a previous review critiqued food pricing measures for relevance specific to a rural context, our review includes both rural and urban contexts [28]. Another review [23] also describes the components of individual instruments, such as the identification of differently sized ‘food baskets’, ranging between 30 and 200 food items. Such critique was beyond the scope of our research questions.

Despite emerging options for electronic methodologies, the predominance of in person, instore data collection continues, notwithstanding the time-consuming and resource-intensive nature of this method. Studies indicate that these instore instruments can be targeted and applied within multiple contexts, such as rural [9–12], Indigenous [129, 130], and low socioeconomic areas [85]. Perhaps researchers consider instore data collection as providing real-world insights at a community and population health level. Our review identified that food pricing instruments were mostly used to monitor food prices at a single point in time (cross-sectional) rather than changes at different time points (longitudinal). Instruments that enable the comparison of food prices in terms of a healthy diet (as recommended by dietary guidelines) compared with current dietary patterns (as reported through population health surveys) [128], and relative affordability for families, appear to provide data of greater practice and policy relevance with regard to community strategies, taxes, and subsidies that have potential to enhance food affordability, availability, and accessibility.

Technological innovations are an emerging alternative to in person data collection, facilitating the acquisition of online supermarket prices, a less labour-intensive method for capturing food prices [131]. To date, this method has been used within major chain-supermarkets, with a recent study reporting similar results when comparing pricing data obtained instore versus online [94]. This method therefore holds potential where an online supermarket presence exists, which was increasingly the case during the COVID-19 pandemic [53], providing rapid feedback to inform price promotions. However, for smaller and/or independent food retail outlets, frequently located in rural areas, online data collection does not appear to capture the contextual nuances of instore price promotions.

Our review found an over-representation of food pricing studies within urban areas. This is consistent with multiple studies that reflect inequities experienced within rural environments [132], and rural *food* environments are no exception [133]. The predominance of research within urban areas may also reflect a pragmatic researcher response to the physical proximity of stores (ease of measurement) and larger population reach (potential for greater population impact). Previous research shows significant differences in income-based variables, food environments, and the affordability of healthy food between urban and rural settings [134]. There is therefore a need for rural-specific food pricing studies, using appropriate instruments, to evaluate and inform rural-specific food environment initiatives [28].

During the period covered by this review, high level experts from the World Health Organization [135], the Lancet Commission [136], and the Food and Agricultural Organisation of the United Nations [137] have identified the potential benefits that initiatives located within food retail environments can provide in nudging dietary choices towards healthier options through instore food pricing and promotion, with the overall aim of improving population level diets [14]. Measures of food pricing, and the relative affordability of a healthy diet, are important to both inform and measure the effectiveness of such initiatives. However, few studies in our review explicitly aimed to inform initiatives or strategies, either at the community or policy level. Assessment of author-reported strengths and limitations of food pricing instruments and methodologies also identified a need for a universal instrument that reflects contextual geographic and socio-cultural information; is intended to be used repeatedly over time; and is adaptable to different country/cultural/contextual settings [17, 23]. Future research would benefit from linking the purpose of undertaking food pricing data collection more explicitly to potential initiatives. Our review supports this call and suggests that the instrument selected should suit the context and collect longitudinal data to provide greater insights into the design and effectiveness of initiatives that make healthy food not only affordable but also available and accessible.

### Strengths and Limitations

This systematic review provides a current and comprehensive overview of international food pricing studies across HIC. We acknowledge that while food prices are an important factor influencing food choice, it is only one component of the food environment; however, analysing instruments that assess food acceptability, availability, and accessibility was beyond the scope of this review. This review focused on HIC and a similar review on food pricing studies in low- and middle-income countries would be informative. This review may have missed additional relevant data as it only included English language studies and did not include grey literature or hand searching of reference lists.

## Conclusion

Food security has come under heightened scrutiny given the food supply interruptions experienced worldwide during the COVID-19 pandemic. While studies providing a snapshot of food prices can be useful to identify areas impacted by rising food prices, much of this cross-sectional data is known. This review raises questions regarding the purpose of collecting food price data, and how this data can best be used to inform change through practice and policy strategies. We suggest that longitudinal studies using a consistent methodology, which acknowledges contextual nuances and demonstrates temporal changes in food pricing, are needed to inform and to evaluate community-based or legislative strategies to improve the relative affordability of a healthy diet.

## Supplementary Information

Below is the link to the electronic supplementary material.

Supplementary file1 (PDF 47 kb)

Supplementary file2 (DOCX 39 kb)
