# Supplementary material for: Assessing the Cost of Healthy and Unhealthy Diets: A Systematic Review of Methods
Source: Curr Nutr Rep. 2022 Sep 9;11(4):600–17. doi: 10.1007/s13668-022-00428-x (PMC9461400; doi:10.1007/s13668-022-00428-x)

Assessing the Cost of Healthy and Unhealthy Diets: A Systematic Review of Methods

Current Nutrition Reports

Cherie Russell[1*](ORCID: 0000-0003-1251-4810)(BFood&NutrSc(Hons)), Jillian Whelan[2] PhD (ORCID: 000000019434109X), Penelope Love[1,3] PhD(Nutr&Diet) (ORCID: 0000-0002-1244-3947)

1. *School of Exercise and Nutrition Sciences, Deakin University, Geelong, Australia*
2. *School of Medicine, Institute for Health Transformation, Deakin University, Geelong, Australia*
3. *Institute for Physical Activity and Nutrition, Deakin University, Geelong, Australia*

Corresponding Author: Cherie Russell, 221 Burwood Highway, Burwood, Australia; email: caru@deakin.edu.au; phone: 0432 313 937

Supplement 1: *PRISMA diagram of literature search*

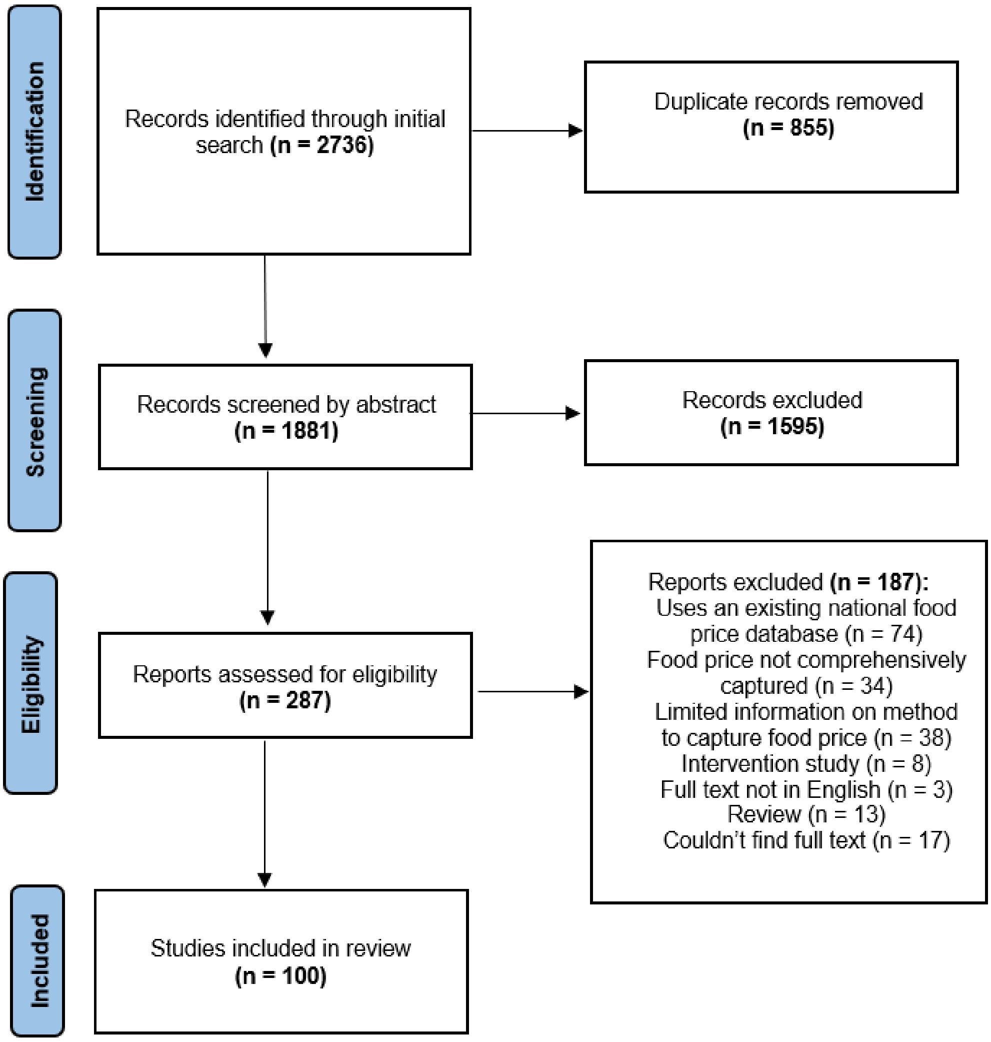

Supplement: Supplementary file 1 — Supplementary file1 (PDF 47 kb) [file 13668_2022_428_MOESM1_ESM.pdf]
