# Supplementary material for: Assessing the Cost of Healthy and Unhealthy Diets: A Systematic Review of Methods
Source: Curr Nutr Rep. 2022 Sep 9;11(4):600–17. doi: 10.1007/s13668-022-00428-x (PMC9461400; doi:10.1007/s13668-022-00428-x)
Supplement: Supplementary file 2 — Supplementary file2 (DOCX 39 kb) [file 13668_2022_428_MOESM2_ESM.docx]

**Assessing the Cost of Healthy and Unhealthy Diets: A Systematic Review of Methods**

**Current Nutrition Reports**

Cherie Russell^1*^(ORCID: 0000-0003-1251-4810)(BFood&NutrSc(Hons)), Jillian Whelan^2^ PhD (ORCID: 000000019434109X), Penelope Love^1,3^ PhD(Nutr&Diet) (ORCID: 0000-0002-1244-3947)

1. *School of Exercise and Nutrition Sciences, Deakin University, Geelong, Australia*
2. *School of Medicine, Institute for Health Transformation, Deakin University, Geelong, Australia*
3. *Institute for Physical Activity and Nutrition, Deakin University, Geelong, Australia*

**Corresponding Author:** Cherie Russell, 221 Burwood Highway, Burwood, Australia; email: caru@deakin.edu.au; phone: 0432 313 937

***Supplement 2:*** *Strengths and limitations, as described by study authors, of commonly used food pricing instruments*

| **Named Instrument** | **Strengths** | **Limitations** | **Study references** |
| --- | --- | --- | --- |
| Victorian Healthy Food Basket (Australia) | - Uses four distinct reference families  - Uses Nutrient Reference Values to assess nutritional adequacy  - Observer bias minimised by using pre-defined product brands and packaging sizes | - Doesn’t reflect actual purchases or consumption as it is a ‘hypothetical’ basket  - Can’t be generalised to other locations or cultural settings  - Cross-sectional; doesn’t capture changes over time or seasonality | Palermo ’16 [19]  Cuttler ’19 [80]  Palermo ’08 [87]  Ward ’12 [91] |
| Healthy Food Access Basket Survey (Australia) | - None specified | - Doesn’t align with the ADGs | Harrison ’07 [83]  Pollard ’14 [88] |
| Adelaide Healthy Food Basket (Australia) | - None specified | - Food markets not captured  - Doesn’t account for wastage  - Doesn’t capture changes over time or seasonality | Tsang ’07 [89]  Wong ’11 [92] |
| The Illawarra Healthy Food Basket Survey (Australia) | - Observer bias minimised by using pre-defined product brands and packaging sizes | - Uses one standard reference family to calculate food affordability, may have limited generalization to a wider population  - Cross-sectional; doesn’t capture changes over time or seasonality  - Food markets not captured  - Doesn’t account for wastage | Tsang ’07 [89]  Walton ’21 [90] |
| Market Basket Survey conducted by the NT Government (Australia) | - Recording instore purchases captures actual purchasing behaviour | - Results not generalisable to other locations or cultural settings  - Online supermarket prices likely to moderately underestimate the price disparity between remote store and in-store supermarket purchases | Ferguson ’16 [81] |
| Food Basket informed by the INFORMAS Framework (Australia) | - Compared the cost of a hypothetical healthy diet and a current diet  - Healthy diet was developed to meet food-based dietary guidelines and Nutrient Reference Values  - Energy requirement for the current diet reflects actual BMI of the population rather than reported energy intake | - Current (unhealthy) diet may not be the same as actual expenditure on food, given that it is based on national mean dietary intakes  - Other healthy menus could fit the food-based dietary guidelines and recommended dietary intake  - Wastage not captured  - The nutrient intake of the current diet was based on older nutrition surveys. may not reflect the nutrient intake of the current diet  - Only one healthy and one current diet was developed for each population group  - Other inputs to the cost of producing a household meal, aside from food prices, not captured  - Doesn’t capture actual household expenditure  - Arbitrary decision points occur around sampling frameworks, data collection protocols, analysis and presentation of results, data sources and definitions of family and household income and composition | Lee ’16 [20]  Mackay ’18 [110] |
| Healthy Diets Australian Standardised Affordability and Price Survey (Australia) | - Recommended diet aligns with the Australian dietary guidelines  - Current diet based on actual consumption  - Practical and time efficient  - Allowed for alternate brands/sizes to be included  - Alcohol included in the survey  - Uses universal sizes so that results are comparable  - Use can be streamlined through application of technologies such as electronic data collection and/or data scraping | - Doesn’t account for out-shopping  - Limited to the reference family used to inform current and recommended diets  - The use of average prices for missing/unavailable items may have led to an underestimation of the cost  - Doesn’t capture changes over time or seasonality  - Doesn’t capture community garden, food swaps, the food pantry or food banks  - Doesn’t capture food waste  - Assumes food is shared equitably by members of the household  - No adjustments were made for costs such as transport, time, cooking equipment and utilities  - True costs of recommended diet likely higher than reported, as diet is modelled for the shortest and least active members in each age and gender group  - No adjustments were made to account for the under-reporting in the AHS 2011–12 | Love ’18 [9]  Lee ’21 [85]  Lee ’20 [86]  Zorbas ’21 [94] |
| Nutrition Environment Measures Survey – Stores (NEMS-S) [including NEMS-S-Rev, TxNEAS and NEMS-S-NL, Bridging the Gap] (USA) | - Studied low prices in a range that reflects discounting levels observed in supermarkets  - Allows for the comparison of food prices between healthy and regular options  - Encourages reporting of reliability  - Peer-reviewed with strong inter-rater and test-re-test reliability  - Validated in multiple countries  - Discounts and promotions captured in some variations  - Store audits described as ‘objective’ | - Doesn’t include some culturally appreciated foods important to specific regions without variation  - Products audited are only a few of the many of potential interest  - Can’t be generalised to other locations or cultural settings  - Lengthy, requires a lot of time to complete  - Results could differ depending on who is administering the survey  - Actual food being purchased was not captured  - Healthiness not confirmed as food is not nutritionally assessed without a variation  - Doesn’t capture changes over time or seasonality  - Need to assess either many/all stores in an area  - Doesn’t include an absolute measure of food affordability  - Some components of the food environment are not included  - Doesn’t account for outshopping  - Low convergent validity between the NEMS-S and perceptions of affordability  - No data on price variability due to specials/discounts | Whelan ’18 [10]  Pereira ’14 [11]  Andreyeva ’08 [33]  Borja ’19 [36]  DiSantis ’14 [44]  Ghosh-Dastidar ’14 [47]  Ghosh-Dastidar ’17 [48]  Jin ’21 [54]  Ko ’18 [56]  Lee Smith ’13 [57]  Shen ’19 [68]  Stroebele-Benschop ’20 [71]  Mah ’20 [106]  Minaker ’13 [107]  Minaker ’14 [108] |
| University of Washington’s Center for Public Health Nutrition (CPHN) Market Basket (USA) | - Captures foods recommended for health using the US Dietary Guidelines  - Validated based on the same method the US Bureau of Labor and Statistics | - Doesn’t capture geographical source of foods which may impact price  - No data on price variability due to specials/discounts  - Data were collected on the lowest priced item available rather than tracking the same brand  - Informal food markets not captured  - Prepared food not captured  - Doesn’t capture purchasing habits  - Results not generalisable to other locations cultural settings  - Doesn’t capture changes over time or seasonality | Buszkiewicz ’19 [38]  Otten ’17 [66]  Spoden ’18 [70] |
| USDA Market Basket (USA) | - None specified | - None specified | Hardin-Fanning ’15 [50]  Harding-Fanning ’17 [51] |
| Market basket developed by Fred Hutchinson Cancer Research Center (USA) | - None specified | - Only reflects prices listed at time of audit  - Results not generalisable to other locations or cultural settings  - Originated from a food frequency questionnaire, thus was limited in its representation of many foods that are commonly consumed  - Cross-sectional; doesn’t capture changes over time or seasonality  -Didn’t measure specials/discounts on foods | Monsivais ’07 [62]  Monsivais ’10 [63]  Monsiviais ’13 [64] |
| Thrifty Food Plan Market Basket (USA) | - None specified | - May not reflect the typical U.S. food preferences or eating habits  - Doesn’t capture other costs associated with preparing food  - Results not generalisable to other locations or cultural settings | Bronchetti ’19 [37]  Christensen ’20 [40]  Greenberg ’20 [49]  Franzen ’10 [46]  Hilbert ’14 [52]  Richards ’06 [67] |
| Revised Northern Food Basket (Canada) | - The diet meets most nutrient requirements and food serving recommendations for Canadians  - Culturally specific  - Choice of foods based on the Australian Guide to Healthy Eating  - Usefulness confirmed by field trials | - Doesn’t include prepared/convenience foods or foods of little nutritional value  - Doesn’t consider out-shopping  - Doesn’t capture actual purchasing behaviours  -Used the lowest cost item available in the defined product volume, thus may represent a lower-end estimate of actual food expenditure  - Basing nutrient profiles on 100kcal favours low energy density foods | Kenny ’18 [103] |
| Unspecified Food Basket (Canada) | - None specified | - Doesn’t capture changes over time or seasonality | Lear ’13 [105] |
| Ontario Nutritious Food Basket (Canada) | - None specified | - None specified | Latham ’07 [104] |
